# Hybrid SEM-ANN model for predicting undergraduates’ e-learning continuance intention based on perceived educational and emotional support

**DOI:** 10.1371/journal.pone.0308630

**Published:** 2024-12-13

**Authors:** Shanshan Xu, Yangxin Wang, Wenbo Luo

**Affiliations:** 1 School of Fine Arts, Yuzhang Normal University, Nanchang, Jiangxi, China; 2 School of Fine Arts, Jiangxi Normal University, Nanchang, Jiangxi, China; Al-Ahliyya Amman University, JORDAN

## Abstract

Based on the Expectation Confirmation Model (ECM), this study explores the impact of perceived educational and emotional support on university students’ continuance intention to engage in e-learning. Researchers conducted a survey using structured questionnaires among 368 university students from three universities in Jiangxi Province. They measured their self-reported responses on six constructs: perceived educational support, perceived emotional support, perceived usefulness, confirmation, satisfaction, and continuance intention. The relationships between predictors and continuance intention, characterized by non-compensatory and non-linear dynamics, were analyzed using Structural Equation Modeling combined with Artificial Neural Networks. Apart from the direct effects of perceived educational and emotional support on perceived usefulness being non-significant, all other hypotheses were confirmed. Furthermore, according to the normalized importance derived from the multilayer perceptron analysis, satisfaction was identified as the most critical predictor (100%), followed by confirmation (29.9%), perceived usefulness (28.3%), perceived educational support (22.6%), and perceived emotional support (21.6%). These constructs explained 62.1% of the total variance in the students’ continuance intention to engage in e-learning. This study utilized a two-stage analytical approach, enhancing the depth and accuracy of data processing and expanding the methodological scope of research in educational technology. The findings of this study contribute to the United Nations’ Sustainable Development Goal 4, which aims to ensure inclusive and equitable quality education and promote lifelong learning opportunities for all by 2030. It provides direction for future research in different environmental and cultural contexts.

## 1. Introduction

E-learning refers to an educational model that delivers content and supports the learning process through digital technology and the Internet outside of traditional classroom settings [1]. This learning mode is widely welcomed for its flexibility, convenience, and abundance of resources [2]. E-learning is not merely a supplement to traditional classroom teaching; it revolutionizes the delivery of education, offering learners the opportunity to access knowledge anytime and anywhere [3]. With the rapid advancement of information technology, the e-learning market has expanded rapidly worldwide. According to Statista [4], the global e-learning market size exceeded $300 billion in 2023 and is expected to reach nearly $400 billion by 2026. Additionally, e-learning has been widely applied across various educational stages and scenarios, such as higher education [5], vocational training [6], and K-12 education [7].

Analyzing students’ motivations for the continuous use of e-learning systems is crucial for educational developers, as it not only provides a strategic advantage in the global market but also helps educators and providers formulate more effective educational and marketing strategies, thereby increasing the actual usage frequency of e-learning systems [8]. Currently, research on e-learning systems primarily focuses on system adoption [9], e-learning policies [10], training [11], and evaluation [8]. Higher education institutions are increasingly recognizing and adopting e-learning as a core learning tool [3, 12]. However, compared to traditional classroom teaching, the lack of direct face-to-face interaction in e-learning environments leads to learner isolation, lack of motivation, and even emotional disconnection [13]. Consequently, the importance of emotional support and social interaction has become increasingly prominent and cannot be ignored.

By providing an encouraging and inclusive learning environment, emotional support can enhance students’ confidence, boost their learning motivation, and increase their persistence [14]. For instance, when students encounter challenges or difficulties in e-learning, timely emotional feedback and encouragement can help them overcome setbacks and maintain a positive learning attitude [15]. Studies have also shown that regular online interactions and feedback can help students build a stronger sense of community belonging, improve their course satisfaction, and enhance learning outcomes and completion rates [16]. Moreover, providing emotional support can reduce loneliness and isolation in online learning environments, directly influencing students’ course loyalty and engagement [17].

In summary, the existing literature provides a multidimensional perspective on understanding the continuance intention (CI) of university students in e-learning environments. However, some gaps have still been observed in the research. First, from a theoretical standpoint, prior studies on CI in e-learning often overlooked the emotional support for students. Compared to traditional classrooms, the e-learning environment, due to the absence of face-to-face direct and intimate interactions, might induce feelings of isolation, lack of motivation, and emotional detachment in learners. Hence, providing emotional support and fostering social interaction is increasingly recognized alongside continuously optimizing instructional content and technology. Secondly, from a methodological perspective, while Structural Equation Modeling (SEM) has been widely used for exploring relationships [18], it has been proven that this model only identifies linear relationships and is unsuitable for non-linear and non-compensatory relationships between exogenous and endogenous constructs. On this basis, Artificial Neural Networks (ANN) algorithms have become an ideal choice for analysis and prediction due to their excellent data adaptability, capability to analyze complex non-linear relationships, and high accuracy and robustness in a multivariate context.

In summary, this study aims to achieve two objectives: (1) In the first phase, to reveal how perceived educational support (PEDS) and perceived emotional support (PEMS) influence undergraduates’ CI to engage in e-learning through perceived usefulness (PU), confirmation (CON), and satisfaction (SAT) using SEM; (2) In the second phase, to develop a high-performance predictive model of undergraduates’ CI in e-learning based on ANN. These findings will provide theoretical and methodological support for interventions in undergraduates’ e-learning while fostering the sustainability and academic achievement of online learning. Accordingly, the research questions for this study are:

1. What factors influence the CI of undergraduates in e-learning?
2. To what extent do these factors explain the variance in the CI of undergraduates in e-learning?
3. What is the normalized importance of the factors affecting the CI of undergraduates in e-learning?

Compared to existing studies, this research contributes in four ways: First, it uncovers the impact of PEMS on the CI of undergraduates in e-learning, an aspect currently overlooked in the literature, thus filling a gap. Second, the study employs an SEM-ANN approach to capture both linear and non-linear, non-compensatory relationships between exogenous and endogenous constructs, better explaining the complexity involved in students’ CI in e-learning. Third, by integrating PEMS and PEDS, the study expands the Expectation Confirmation Model (ECM), enabling a better understanding of the underlying mechanisms affecting students’ CI in e-learning. Finally, this study addresses the United Nations’ Sustainable Development Goal (SDG) 4, which aims to ensure inclusive and equitable quality education and promote lifelong learning opportunities for all by 2030. By examining the factors influencing university students’ CI to engage in e-learning, this research provides insights that can help improve educational practices and policies, thereby contributing to sustainable educational development.

The rest of this paper is organized as follows: The second section is a literature review; the third section introduces the theoretical model of the study and hypothesizes the relationships between constructs; the fourth section describes the methodology and findings of the study; the fifth section discusses the results; finally, we discuss the limitations of the research, future directions, and conclusions.

## 2. Literature review and hypothesis development

### 2.1 ECM

The ECM is based on the Expectation Confirmation Theory and the Technology Acceptance Model (TAM) [19]. This model encompasses four key constructs: PU, SAT, CON, and CI [19]. PU describes users’ perceptions of the system’s ability to enhance their work or study efficiency; SAT refers to users’ subjective evaluation of their overall experience after using a product or service; CON is the perception of how well the actual performance of an information system matches user expectations; and CI denotes the tendency of users to keep using the system.

Extensive research using ECM in the e-learning domain has been conducted. The contexts of existing studies include online learning [20], online learning systems [21, 22], blended learning [23], learning management systems [24], and MOOCs [25]. Based on the ECM and an integrated model of the TAM with social influences and perceived enjoyment, Ashrafi examined the factors influencing the CI of 153 university students using a learning management system [24]. Results indicated that PU strongly predicted students’ CI. Additionally, external variables related to ECM include curiosity and attitude [25], subjective norms [22], perceived enjoyment [24], extrinsic and intrinsic motivation [26], and self-efficacy [23]. Finally, scholars have also attempted to integrate ECM with other models such as TAM [24], Theory of Planned Behavior [20], Information System Success Model [27], and Self-Determination Theory [26] to form new models aimed at more deeply explaining the factors influencing CI. Based on these studies, this paper employs ECM to explore the factors affecting the CI of university students in e-learning. Yang integrated intrinsic motivation and academic self-efficacy into the ECM and the Information System Success Model to investigate the factors influencing the CI of 1845 novice blended learners [27]. The results showed that performance expectations, intrinsic motivation, and SAT significantly positively affected the CI of novice blended learners.

### 2.2 Hypothesis development

#### 2.2.1 PEDS

PEDS has increasingly become a central topic in educational research. It refers to students’ belief that educators, such as teachers and mentors, provide resources and assistance that facilitate academic success [28]. This type of support involves offering necessary learning resources, strategies, and feedback through specific academic assistance [28]. Previous research has highlighted numerous benefits of educational support in e-learning environments [29, 30]. These advantages include but are not limited to accommodating learners’ differences [31], emphasizing students’ autonomous learning [32], reducing the limitations of traditional face-to-face teaching [33], and increasing interactions between students and experts [34].

When students PEDS (e.g., explanations of issues by teachers), they are more likely to actively engage in learning and develop learning skills, thereby enhancing the perceived educational value. Consequently, numerous studies have demonstrated that PEDS positively influences PU [15, 28]. For instance, within the framework of the TAM combined with social theory, He et al. found that university students’ PEDS significantly positively affected their PU in the context of online learning CI [15]. Moreover, researchers have found that PEDS can significantly and positively influence students’ CON of e-learning [35, 36]. For example, Maheshwari and Kha explored the relationship between educational support and entrepreneurial intentions among Vietnamese university students, discovering that when students PEDS, they showed higher levels of CON towards their entrepreneurial intentions [36]. Based on these findings, this study proposes the following hypotheses:

H1: University students’ PEDS significantly impacts PU in e-learning.
H2: University students’ PEDS significantly impacts CON in e-learning.

#### 2.2.2 PEMS

PEMS has increasingly garnered attention in psychological health and education. It refers to students feeling that their emotional needs are understood, cared for, and respected [28]. This type of support can be understood as providing compassion, friendliness, encouragement, respect, and care, all of which are non-academic [28]. Furthermore, emotional support plays a crucial role in learning [37] as it can encourage and comfort learners, enhancing their well-being when facing negative emotions.

In recent years, emotional support has increasingly been the focus of researchers’ attention [38]. On one hand, many scholars have revealed the importance of emotional support in students’ e-learning. According to the theory of emotional regulation, core components of PEMS include emotional recognition, self-awareness, and social support, among others researcher [39]. O’regan focused on the close connection between emotions and students’ e-learning, emphasizing the significant role specific emotions play for students [40]. Additionally, Anderson* and others believed that interactions and emotional support among learning community members can help them achieve their learning objectives [41].

On the other hand, researchers have also delved into the relationship between emotional support and educators. Indeed, as modern societal pressures increase and mental health awareness improves, emotional support and management have become particularly crucial in key areas such as education and career development. During this time, positive and negative emotions can significantly impact educational workers’ transformation [42]. Moreover, through emotional support, educators can provide students with key strategies to optimize classroom teaching outcomes [43]. Overall, PEMS has become an indispensable area of research and application in psychological health and education.

Existing research indicates that adequate emotional support can reduce the psychological burden required to manage negative emotions, lessen the challenges associated with adapting to e-learning, and thereby enhance learning efficiency and outcomes, as well as strengthen its PU [44, 45]. For instance, C. Li et al. explored the factors influencing CI in online health communities from a social support perspective, finding that users’ PEMS significantly positively affected their PU [44]. Additionally, researchers have found that PEMS can significantly positively influence students’ level of CON [38, 46, 47]. For example, based on Expectation Confirmation Theory, Liu and Wang studied the factors influencing the CI of using online mental health communities, and the results indicated that when users felt emotional support, they showed a higher level of CON towards the community platform [38]. Based on these findings, this study proposes the following hypotheses:

H3: University students’ PEMS significantly impacts PU in e-learning.
H4: University students’ PEMS significantly impacts CON in e-learning.

#### 2.2.3 CON, PU, and SAT

When university students use e-learning tools and resources and find that these meet their learning needs and expectations, they are more likely to perceive the usefulness of e-learning [17, 47]. For instance, Li used an extended ECM to explore the factors influencing the CI of Chinese English learners using automated writing evaluation tools, finding that CON directly impacts learners’ PU of these tools [47]. Zhang et al. incorporated the variables of time distortion and focused attention into the ECM to study the SAT and CI of Chinese university students using virtual and remote laboratories, discovering that higher levels of student CON are associated with increased PU [17].

Furthermore, when university students’ expectations about e-learning are confirmed, they are more likely to feel satisfied with this learning mode and will continue using it. Thus, CON can also significantly affect students’ SAT with e-learning [48, 49]. Sasono and Pramana examined users’ CI after implementing gamification in e-learning, finding that an increase in CON among gamified users also increased their SAT with e-learning [48]. Based on these findings, this study proposes the following hypotheses:

H5: University students’ CON significantly impacts PU in e-learning.
H6: University students’ CON significantly impacts SAT in e-learning.

#### 2.2.4 PU, SAT, and CI

Numerous studies indicate that PU is a critical predictor of students’ SAT with e-learning systems [50, 51]. For example, Al-Hamad et al. combined the ECM with the TAM to explore the impact of fear during the COVID-19 pandemic on the technology adoption of teachers and students [50]. Their findings showed that the higher the students’ PU, the more satisfied they were with mobile learning platforms.

When university students perceive that e-learning tools and resources help them achieve their learning goals more effectively, they are likelier to continue using them. Therefore, PU can significantly influence students’ CI to use e-learning systems [48, 52]. Using the Expectation Confirmation Theory, Sasono and Pramana investigated the impact of gamification in e-learning on users’ CI to use, revealing that higher PU led to a greater likelihood of continued use of the e-learning system [48]. Based on a modified UTAUT model, Widjaja and Widjaja explored the factors affecting graduate students’ intentions to use digital libraries. They found that PU was a key predictor of their CI [52]. Based on these studies, this research proposes the following hypotheses:

H7: University students’ PU significantly impacts SAT in e-learning.
H8: University students’ PU significantly impacts CI in e-learning.

#### 2.2.5 SAT and CI

When university students feel highly satisfied with the e-learning tools and resources they use, they are more likely to exhibit a CI to engage in e-learning. Numerous studies have confirmed a positive relationship between students’ SAT with e-learning and their CI [48, 53]. Specifically, Puriwat and Tripopsakul investigated the impact of e-learning quality on SAT and CI among higher education students in Thailand [53]. Their findings indicated that students’ SAT significantly positively affected their CI to use e-learning platforms. Similarly, Sasono and Pramana, using the ECM, explored the impact of gamification in e-learning on users’ CI, showing that SAT directly influences users’ CI to engage in e-learning [48]. This study posited that the higher the university students’ SAT with e-learning, the stronger their CI will be. Based on existing research, this study proposes the following hypothesis:

H9: University students’ SAT significantly impacts CI in e-learning.

## 3. Methods

### 3.1 Sample and data collection

The researchers confirm that all research was performed in accordance with relevant guidelines/regulations applicable when human participants are involved (e.g., Declaration of Helsinki or similar). This study was approved by the Ethics Committee of Jiangxi Normal University, with the approval number JNU-2023-05-0013. This study collected data and tested the proposed hypotheses using the online survey platform Sojump (www.Sojump.com). The data collection period spanned from October 12 to December 21, 2023. Prior to the survey, participants were fully informed about the study’s purpose and assured that participation was entirely voluntary and that their personal information would be strictly protected. All participants provided informed consent in the form of an electronic signature.

To ensure the diversity and representativeness of the sample, this study selected three universities in Jiangxi Province, representing different types of institutions and covering a variety of academic disciplines, including humanities, sciences, and engineering. A total of 368 valid questionnaires were collected, including 71 males (19.3%) and 297 females (80.7%). The majority of participants were aged between 18 and 20 years (79.3%), with 16.6% under 18, 3.5% between 20 and 22, and 0.6% aged 22 and above. By grade level, the sample included 347 freshmen (94.3%), three sophomores (0.8%), ten juniors (2.7%), and eight seniors (2.2%). Participants’ academic backgrounds were distributed among the humanities (65.5%), social sciences (33.9%), and natural sciences (0.6%) (Table 1).

**Table 1:** Demographic characteristics of the sample (N = 368).

Demographic characteristics	Categories	Quantities	Percentage
**Genders**	Male	71	19.3%
	Female	297	80.7%
**Age**	Under 18	61	16.6%
	18–20 years	292	79.3%
	20–22 years	13	3.5%
	22 years and over	2	0.6%
**Grade**	Freshman	347	92.3%
	Sophomore	3	0.8%
	Junior	10	2.7%
	Senior	8	4.2%
**Professional category**	Humanities	241	65.5%
	Social sciences	125	33.9%
	Natural sciences	2	0.6%

The study employed random sampling techniques to select students from the three universities in Jiangxi Province. Random sampling helps reduce selection bias, increasing the representativeness of the sample and the generalizability of the study results. Specifically, within each university, the research team generated a random sampling list based on student ID numbers and sent survey invitations through the university’s email system. To enhance the response rate, the research team also posted the survey link on social media platforms commonly used by university students, such as WeChat.

In this study, a maximum of three arrows point towards PU. According to Hair et al., with a significance level of 5%, the R^2^ should exceed 10%, and the minimum required sample size is 124 [54]. With 368 participants, this study meets the minimum statistical requirements. Additionally, to ensure the representativeness and robustness of the results, the study conducted a chi-square test for gender representativeness (male = 43.23%, female = 50.62%; p = 0.518). The results indicated no significant difference between the sample and the overall distribution, further verifying the high representativeness of the sample.

### 3.2 Instruments

The questionnaire survey comprised two parts: The first part gathered participants’ demographic information. The second part addressed the constructs involved in this study. Variables were assessed using established scales, which were appropriately modified to fit the context and objectives of the study. In comparison to the Likert seven-point scale, the Likert five-point scale is easier to understand [55], simpler to analyze [56], more adaptable [57], and demonstrates higher reliability and validity [58]. Therefore, each construct was measured using a Likert five-point scale, ranging from (1) “Strongly Disagree” to (5) “Strongly Agree.” S1 Appendix provides a detailed list of the scales used in this study.

### 3.3 Data analysis

This study employs a two-stage approach to test the hypotheses and develop the predictive model. First, SEM is used to identify the linear relationships between exogenous and endogenous variables. SEM is effective in detecting these linear relationships, as discussed in recent studies by Wang et al. [59], Zhang and Li [60], and Liu et al. [61]. However, SEM has limitations in capturing nonlinear and non-compensatory relationships, which are crucial for understanding the complex dynamics in e-learning contexts.

To address these limitations, the second stage of this study integrates ANN. ANN can capture both linear and nonlinear relationships using non-compensatory models, thereby improving predictive accuracy. Recent studies by Zhang et al. [62], Chen et al. [63], and Li and Zhao [64] emphasize the robustness of ANN in handling complex data patterns and its applicability in predictive tasks. By combining SEM and ANN, this study enhances the depth and accuracy of data analysis, providing a comprehensive understanding of the factors influencing students’ CI in e-learning.

## 4. Results

### 4.1 Measurement model

The assessment of the measurement model required testing the reliability and validity of the questionnaire and data. Reliability was evaluated by examining the factor loadings for each variable. Factor loading scores above 0.70 indicate high reliability [65]. According to the results in Table 2, all item factor loadings exceeded 0.70, indicating they are highly reliable.

**Table 2:** Reliability and validity.

Constructs	Items	Loadings	Cronbach’s alpha	CR	AVE
CI	CI _1	0.907	0.905	0.942	0.844
	CI _2	0.945			
	CI _3	0.903			
CON	CON _1	0.907	0.904	0.939	0.837
	CON _2	0.934			
	CON _3	0.904			
PEDS	PEDS _1	0.753	0.817	0.884	0.658
	PEDS_2	0.878			
	PEDS _3	0.873			
	PEDS_4	0.728			
PEMS	PEMS _1	0.837	0.876	0.916	0.732
	PEMS _2	0.891			
	PEMS _3	0.83			
	PEMS_4	0.862			
PU	PU _1	0.926	0.901	0.943	0.847
	PU _2	0.927			
	PU _3	0.907			
SAT	SAT _1	0.901	0.908	0.944	0.850
	SAT _2	0.945			
	SAT _3	0.919			

Internal consistency was assessed using composite reliability and Cronbach’s alpha coefficients. In this study, Cronbach’s alpha values ranged from 0.817 to 0.908, meeting the acceptable standard (a threshold of 0.7 is considered acceptable). Additionally, according to Hair, composite reliability values between 0.60 and 0.70 are considered acceptable, and values between 0.70 and 0.90 are generally considered satisfactory [54]. In this study, all items’ composite reliability values ranged from 0.884 to 0.944, which meets the above standards.

Convergent validity was measured through the average variance extracted (AVE). In this study, the AVE values ranged from 0.658 to 0.850, exceeding the threshold of 0.5. According to Henseler, this range is acceptable, indicating that the results passed the test for convergent validity [66].

Discriminant validity refers to the extent to which a construct is distinct from other constructs within a model [67]. The Fornell-Larcker criterion is used to assess discriminant validity [68]. According to this criterion, the condition for establishing discriminant validity is that the square root of the average variance extracted (AVE) for each construct should be greater than the correlation coefficient between that construct and any other construct [66]. Based on the results presented in Table 3, the square root of the AVE for each construct exceeded its highest correlation with any other construct. Thus, the results meet the requirements for discriminant validity, indicating that each construct in the study is adequately distinct from the others, thereby validating the individual identity and contribution of each construct within the overall model.

**Table 3:** Fornell-Larcker criteria.

	CI	CON	PEDS	PEMS	PU	SAT
CI	**0.919**					
CON	0.701	**0.915**				
PEDS	0.484	0.515	**0.811**			
PEMS	0.492	0.544	0.806	**0.856**		
PU	0.682	0.755	0.548	0.578	**0.920**	
SAT	0.784	0.811	0.514	0.538	0.808	**0.922**

Note: Data bolded on the diagonal is the square root of AVE

According to the cross-loading criterion, an indicator’s outer loading on its associated construct should be greater than any of its cross-loadings (i.e., its correlations) on other constructs [54]. Table 4 shows that the outer loadings for all constructs are greater than their correlations with any other constructs. This result further supports discriminant validity across all constructs in this study. This finding signifies that each indicator is more strongly associated with its construct than any other, reinforcing the distinctiveness of each construct within the model. Such a result is crucial for ensuring the integrity and interpretability of the model, as it confirms that the constructs are not only statistically distinct but also meaningfully different in terms of what they measure. This enhances the credibility of the study’s findings and the theoretical implications drawn from the data.

**Table 4:** Discriminant validity—Cross loadings.

	CI	CON	PEDS	PEMS	PU	SAT
CI _1	**0.907**	0.664	0.451	0.449	0.668	0.773
CI _2	**0.945**	0.651	0.458	0.468	0.611	0.722
CI _3	**0.903**	0.612	0.422	0.435	0.591	0.652
CON _1	0.647	**0.907**	0.448	0.483	0.715	0.703
CON _2	0.621	**0.934**	0.488	0.484	0.663	0.718
CON _3	0.655	**0.904**	0.477	0.521	0.695	0.803
PEDS _1	0.385	0.345	**0.753**	0.518	0.384	0.382
PEDS _2	0.401	0.473	**0.878**	0.741	0.452	0.448
PEDS _3	0.397	0.461	**0.873**	0.812	0.456	0.437
PEDS _4	0.387	0.382	**0.728**	0.615	0.478	0.397
PEMS_1	0.488	0.502	0.682	**0.837**	0.566	0.516
PEMS_2	0.392	0.474	0.748	**0.891**	0.491	0.461
PEMS_3	0.353	0.384	0.682	**0.83**	0.407	0.343
PEMS_4	0.421	0.472	0.742	**0.862**	0.492	0.495
PU_1	0.635	0.692	0.528	0.565	**0.926**	0.742
PU_2	0.591	0.655	0.494	0.499	**0.927**	0.714
PU_3	0.651	0.741	0.488	0.534	**0.907**	0.778
SAT _1	0.662	0.745	0.477	0.478	0.724	**0.901**
SAT _2	0.742	0.765	0.462	0.502	0.783	**0.945**
SAT _3	0.757	0.737	0.486	0.512	0.735	**0.919**

### 4.2 Structural model

Multiple indicators were employed to analyze the structural model in this study, including tests for multicollinearity, significance testing of path coefficients, and the coefficient of determination (R^2^). These indicators help evaluate the model’s reliability and explanatory power.

#### 4.2.1 Multicollinearity test

According to Hair, a multicollinearity test is recommended to determine any multicollinearity issues within the model [54]. As a rule of thumb, a Variance Inflation Factor (VIF) less than 3.3 indicates excellent values [69], and a VIF less than five is commonly accepted to indicate no concerns of collinearity [54]. Table 5 shows that the VIF values for all variables range from 1.442 to 3.536, indicating that multicollinearity is not likely to affect this study. This absence of multicollinearity suggests that the model’s variables are sufficiently independent to provide reliable and valid results. The VIF values well below the commonly accepted thresholds ensure that the estimated coefficients of the structural model are not biased due to intercorrelations among the predictors, enhancing the robustness of the findings.

**Table 5:** VIF.

	CI	CON	PEDS	PEMS	PU	SAT
CI						
CON					1.442	2.334
PEDS		3.337			3.396	
PEMS		3.337			3.536	
PU	2.901					2.334
SAT	2.901					

#### 4.2.2 Path hypothesis testing

**Fig 1:**
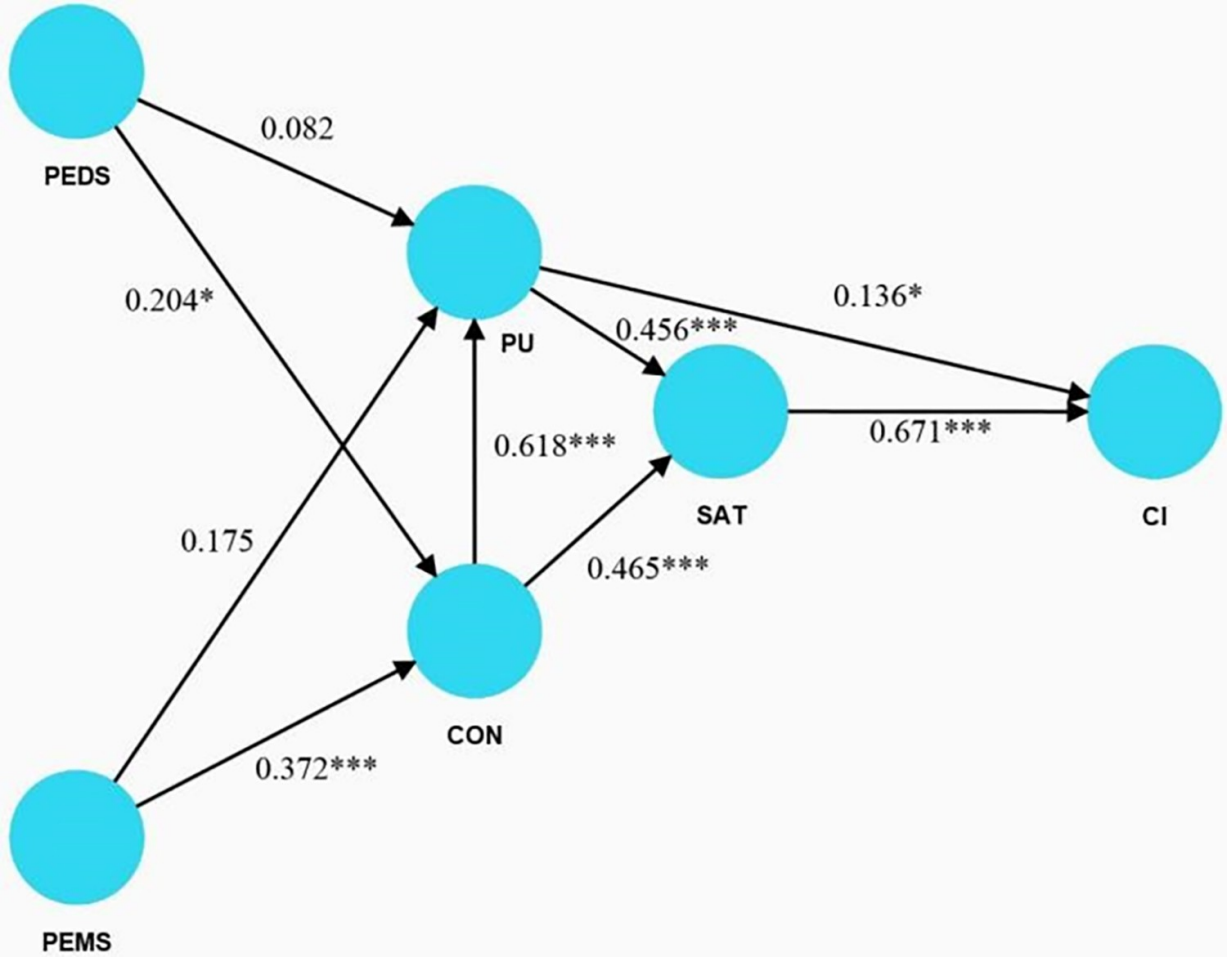
Structural model.

In the structural model, significance tests are used to determine the effects of exogenous variables on endogenous variables (Fig 1). Table 6 indicates significant predictors of CI, where SAT emerged as the strongest predictor (β = 0.671; t = 11.108; p = 0.000), followed by PU (β = 0.136; t = 2.117; p = 0.035). Thus, hypotheses H8 and H9 are supported. In the predictors for SAT, CON was the strongest predictor (β = 0.465; t = 8.915; p = 0.000), followed by PU (β = 0.456; t = 8.531; p = 0.000). Therefore, hypotheses H6 and H7 are supported. PEMS (β = 0.372; t = 3.980; p = 0.000) and PEDS (β = 0.204; t = 2.152; p = 0.030) significantly positively influenced CON. Thus, hypotheses H2 and H4 are supported. CON positively impacted PU (β = 0.618; t = 12.104; p = 0.000), supporting hypothesis H5. However, PEMS (β = 0.175; t = 1.917; p = 0.054) and PEDS (β = 0.082; t = 1.002; p = 0.317) did not significantly affect PU. Therefore, hypotheses H1 and H3 are not supported. These results underscore the complexity and interdependencies within the constructs of this e-learning model. SAT and CON are pivotal in influencing CI and PU, respectively. However, the influence of perceived support on PU did not hold as hypothesized, indicating that factors contributing to PU might be more complex or influenced by other variables not considered in this model.

**Table 6:** Results of path hypothesis testing.

Hypothesis	Relationship	Original sample (O)	T statistics	P values	Results
H1	PEDS →PU	0.082	1.002	0.317	Not Supported
H2	PEDS →CON	0.204	2.152	0.030	Supported
H3	PEMS →PU	0.175	1.917	0.054	Not Supported
H4	PEMS →CON	0.372	3.980	0.000	Supported
H5	CON →PU	0.618	12.104	0.000	Supported
H6	CON →SAT	0.465	8.915	0.000	Supported
H7	PU →SAT	0.456	8.531	0.000	Supported
H8	PU →CI	0.136	2.117	0.035	Supported
H9	SAT →CI	0.671	11.108	0.000	Supported

#### 4.2.3 Coefficient of determination (R^2^)

The coefficient of determination (R^2^) measures the extent to which the independent variables explain the variance in the dependent variable. According to Chin, R^2^ values can be interpreted as strong (0.67), moderate (0.33), and weak (0.19). Table 7 indicates that the R^2^ for CI in e-learning is 0.62, considered moderate to high [70]. This suggests that the model explains 62% of the variance in the dependent variable of CI. This level of R^2^ is quite substantial for social science research, where many potential uncontrollable variables might influence outcomes. An R^2^ value of 0.62 demonstrates that the model captures a significant portion of the factors affecting CI in e-learning, confirming the variables’ relevance and effectiveness in explaining and predicting student behavior in this context. This robustness in explanatory power underscores the strength of the theoretical framework and the operationalization of the constructs within this study.

**Table 7:** Explanatory power of the model.

	R^2^
CI	0.621
CON	0.308
PU	0.615
SAT	0.747

### 4.3 Common method bias

Common method bias refers to the spurious associations among variables that arise in research due to the use of the same method, timing, survey tools, or subjective judgments by the researcher, which might interfere with the accuracy of the findings, making the observed associations appear more significant than they are [71]. Common method bias was assessed using two approaches: Firstly, the Harman single-factor test was conducted to determine whether a single factor accounts for most of the covariance among the variables [72]. The results indicated that the largest single factor explained 26.231% of the variance, which is well below the 50% threshold, suggesting that no single factor dominates the variance explanation in the data. Secondly, the marker variable technique was employed, including a theoretically unrelated marker variable in the research model to test for common method bias [73]. The maximum shared variance with other variables was estimated at 0.0174 (1.74%), which is very low [74]. This indicates minimal interference from common method bias. Based on the results of these two tests, it can be inferred that no significant common method bias is affecting the study. This conclusion enhances confidence in the validity of the relationships identified between the study variables, suggesting that the observed relationships are likely to be genuine and not artifacts of the research methodology.

### 4.4 ANN analysis

In the subsequent phase of this research, similar to the study by Liébana-Cabanillas, the significant factors identified through SEM were used as input neurons in an ANN model [75]. Several factors justified the application of ANN: the data did not follow a normal distribution, and there were non-linear relationships between exogenous and endogenous variables. Furthermore, ANN exhibit robustness against noise, outliers, and smaller sample sizes. They also adapt well to non-compensatory models, where a decrease in one factor does not require compensation by an increase in another. The analysis was conducted using the SPSS Neural Network Module from IBM. ANN can capture linear and non-linear relationships without a normal distribution [76]. The algorithms learn through training, utilizing a Feed-Forward Back-Propagation (FFBP) algorithm for predictive analysis [77]. The input and hidden layers employed multilayer perceptrons and sigmoid activation functions [78]. By iterating through multiple learning rounds, errors are minimized, enhancing the accuracy of predictions [79]. Like Leong, this study used 80% of the sample for training and the remaining for testing [80]. A ten-fold cross-validation process was employed to mitigate the risk of overfitting, yielding Root Mean Square Error (RMSE) values of 0.0819 for training and 0.0828 for testing, indicating a very good model fit [81]. Table 8 shows these RMSE values, confirming the model’s excellent performance.

**Table 8:** Root mean square of error values.

Training			Testing			Total samples
N	SSE	RMSE	N	SSE	RMSE	
294	1.845	0.0779	74	0.616	0.0906	368
300	1.994	0.0819	68	0.682	0.0912	368
296	2.081	0.0843	72	0.702	0.0903	368
291	2.057	0.0827	77	0.414	0.0729	368
301	1.874	0.0789	67	0.535	0.0828	368
300	2.058	0.0826	68	0.423	0.0741	368
296	2.215	0.0868	72	0.576	0.0823	368
288	2.123	0.0825	80	0.488	0.0853	368
301	1.865	0.0786	67	0.624	0.0900	368
293	2.145	0.0839	75	0.362	0.0699	368
Mean	2.0257	0.0819	Mean	0.5422	0.0828	
Sd		0.0028	Sd		0.0081	

Note:N: number of samples; SSE: sum square of error; RMSE: root mean square of error

A sensitivity analysis was conducted to assess the predictive power of each input neuron (as shown in Table 9). This involved obtaining the normalized importance of these neurons by dividing their relative importance by the maximum importance and presenting it in percentage form [82]. The results indicate that SAT is the most critical predictive factor, with a normalized importance of 100%. This is followed by CON, which has a normalized importance of 29.9%, and then PU (28.3%), PEdS (22.6%), and PEmS (21.6%).

**Table 9:** Sensitivity analysis.

ANN	CON	PEDS	PEDS	PU	SAT
ANN 1	0.313	140	0.115	0.102	1.000
ANN 2	0.417	0.406	0.297	0.412	1.000
ANN 3	0.392	0.103	0.178	0.694	1.000
ANN 4	0.186	0.234	0.258	0.221	1.000
ANN 5	0.175	0.216	0.132	0.225	1.000
ANN 6	0.219	0.215	0.222	0.137	1.000
ANN 7	0.536	0.337	0.253	0.411	1.000
ANN 8	0.281	0.241	0.318	0.391	1.000
ANN 9	0.281	0.187	0.191	0.110	1.000
ANN 10	0.179	0.189	0.213	0.113	1.000
Mean importance	0.298	0.227	0.218	0.282	1.000
Normalized importance (%)	29.9%	22.6%	21.6%	28.3%	100.0%

## 5. Discussion

This study aimed to explore the impact of perceived educational and emotional support on the CI to engage in e-learning among undergraduate students. Through a systematic review of existing literature, the study hypothesized that PEDS, PEMS, PU, CON, and SAT play significant roles in influencing the CI of undergraduate students to engage in e-learning. The proposed research framework was assessed using Smart-PLS, with most of the hypotheses being validated, collectively explaining 62.0% of the total variance in students’ CI to engage in e-learning. A detailed discussion of the results concerning the initial research questions will be conducted in subsequent chapters.

Interestingly, PEDS did not significantly impact PU among undergraduate students in their use of e-learning. This finding contrasts with He et al., who found that PEDS positively affected PU among Norwegian high school students [15]. The inconsistency might be attributed to significant differences in learning methods and styles among undergraduate students. Thus, despite educational support, undergraduate students may still choose the most suitable learning methods based on their preferences and needs. Moreover, with the widespread dissemination of e-learning resources and tools, undergraduate students have gradually developed strong self-directed learning capabilities. When assessing the effectiveness of learning tools or resources, they tend to rely more on their own experiences and judgments than external educational support.

In the e-learning context, undergraduate students demonstrated a significant positive impact of PEDS on CON. This aligns with previous findings by Liu et al. [35]. In this study, on the one hand, modern undergraduate students, born in the age of information technology, have developed a strong dependence and identification with e-learning tools due to their daily interactions with technology. The e-learning environment provides immediate feedback, personalized learning paths, and a wealth of resources, making it easier for students to recognize the value and effectiveness of their learning. On the other hand, many e-learning platforms feature social functionalities that enable students to interact and collaborate online with peers or teachers. This interactive form of educational support enhances students’ deep understanding and affirmation of the learning content.

However, PEMS was not found to significantly impact PU among undergraduate students using e-learning. This finding contradicts the research by C. Li et al. [44]. The discrepancy could be attributed to the strong adaptability of modern undergraduate students to digital technologies, which enables them to quickly embrace and integrate new technologies, diminishing the role of emotional support as a primary factor in their learning. Moreover, with the increasing diversity of e-learning resources, undergraduate students enjoy greater autonomy in their choices. Therefore, when emotional support from electronic resources is lacking, they are more likely to seek and switch to other resources that better suit their needs, diminishing their importance on PEMS.

PEMS significantly impacts CON when undergraduate students use e-learning, a result corroborated by Maheshwari and Kha [36]. They observed that when users of online mental health communities feel emotionally supported, they have a higher degree of CON about the community platform. In this study, when undergraduate students perceive emotional support from e-learning tools, they tend to form a more positive emotional connection with the learning content, affirming the content and the learning methods used. Additionally, the need for emotional support may be triggered by the psychological responses of undergraduate students facing learning challenges and difficulties [46]. If students feel that emotional support from e-learning tools helps them more effectively address learning challenges, this positive perception will likely deepen their acceptance of these tools. Thus, the higher the level of emotional support students perceive, the stronger their CON of the e-learning tools.

CON also significantly impacts PU among undergraduate students using e-learning, as validated by Zhang et al. [83]. They found that the higher the degree of CON Chinese undergraduate students have towards virtual and remote laboratories, the higher their PU. E-learning typically includes real-time feedback features. When undergraduate students receive timely feedback and confirm their learning outcomes, this directly enhances the PU of the tools. Furthermore, when e-learning tools meet or exceed students’ expectations, their CON of these tools increases, naturally enhancing their PU. This allows students to focus more on academic content rather than the technical hurdles of learning tools. Therefore, a higher degree of CON among undergraduate students towards e-learning tools correspondingly increases their PU in academics.

CON significantly impacts SAT when undergraduate students use e-learning, aligning with findings from Sasono and Pramana [48]. They noted that CON positively influences teachers’ SAT with using e-learning platforms. In this study, when undergraduate students find that the functionalities of e-learning platforms meet their learning needs and effectively aid their learning process, their sense of CON is strengthened, enhancing their SAT. Moreover, e-learning provides a vivid and convenient learning experience through its interactive elements and immediate access to resources, allowing students to freely adjust their learning paths and pace. CON of this autonomy further enhances their SAT.

Additionally, PU significantly impacts SAT among undergraduate students using e-learning, consistent with the findings of Al-Hamad et al. [50]. They argued that PU significantly and directly impacts learners’ SAT with e-learning systems. E-learning platforms offer many practical features and tools, such as video lectures, interactive quizzes, and forum discussions. When undergraduate students perceive these features as genuinely helpful to their learning process, they tend to be more satisfied with this learning mode. Furthermore, e-learning platforms often provide courses and case studies relevant to real-world applications, enabling students to better integrate what they learn with practical applications. The perception of this practicality enhances students’ SAT.

PU significantly and positively impacts the CI to use e-learning among undergraduate students, corroborating the findings of Widjaja and Widjaja [52]. Their research highlighted that the PU of digital libraries is a crucial predictor of CI among graduate students. In this study, when undergraduate students perceive e-learning tools as useful, they are more likely to engage with and adapt to the technology, further motivating them to use these tools for long-term academic advancement. Additionally, students’ perceptions of the practicality of e-learning tools may be influenced by their focus on self-directed learning and flexibility. If students believe that the tools allow them to plan their learning process more autonomously and flexibly, this positive view could motivate them to use them more extensively. Thus, the stronger the PU of e-learning by undergraduate students, the stronger their motivation to continue using it.

SAT also significantly impacts the CI to use e-learning among undergraduate students, supported by Puriwat and Tripopsakul [53]. They found that the SAT of undergraduate students in higher education in Thailand significantly positively affects their CI to use e-learning platforms. On one hand, the convenience of e-learning platforms saves time for students, enhancing their SAT and thus improving their learning efficiency. This increase in efficiency further enhances their positive intention to continue using such platforms. Additionally, e-learning platforms enable students to instantly understand their learning progress and outcomes. This real-time feedback ignites their enthusiasm for learning; their SAT increases as students feel their growth and progress. Such positive experiences deepen their trust and reliance on the platform, creating a continuous, positive feedback loop.

### 5.1 Theoretical implications

Firstly, this study enhances the comprehensive understanding of the factors influencing the CI to use e-learning among undergraduate students. Specifically, although most research on e-learning emphasizes that educational functionality is the core factor driving its use, this study argues that the e-learning environment should also satisfy students’ psychological needs. Therefore, this research introduced perceived educational and emotional support to examine their impact on the CI to use e-learning. The results indicate that while both forms of support have a significant positive effect on CON, they do not have a noticeable impact on enhancing PU.

Secondly, this study employs the SEM-ANN method to capture the linear-nonlinear and non-compensatory relationships between exogenous and endogenous variables, providing a new methodological perspective for e-learning research. The integration of this method not only enhances the depth and accuracy of data processing and expands the methodological scope of educational technology research. Through this comprehensive approach, understanding of e-learning behavioral patterns is improved, the complexities of undergraduate students’ CI to use e-learning are explained better, and more comprehensive insights for future educational practice and policy-making are provided.

Thirdly, compared to previous literature [15, 44, 84], this study reports contradictory findings, such as the lack of a significant impact of PEDS on PU and the lack of a significant impact of PEMS on PU in e-learning among undergraduate students. This calls for more exhaustive research, necessitating the inclusion of larger, more representative samples.

### 5.2 Practical implications

This study explores the factors influencing undergraduate students’ CI to use e-learning. Based on the findings of this study, the following practical implications are proposed:

Firstly, for educational institutions and educators, by comprehensively exploring how educational and emotional support affect students’ CI to engage in e-learning, a deeper understanding of students’ real needs and preferences in an e-learning environment can be developed. This understanding enables the provision of a richer and more personalized learning experience. The research highlights the significant impact of educational and emotional support on students’ e-learning experiences, suggesting that educational institutions and educators should prioritize professional development and teacher training. This involves improving teachers’ skills in using e-learning tools and fostering their empathy, communication abilities, and student guidance skills. Additionally, it is crucial to provide relevant resources and training that enable teachers to identify and address students’ emotional and learning needs more effectively, thereby creating a more supportive and encouraging learning environment.

Secondly, the findings of this study provide important guidance for developers of e-learning platforms in terms of platform design and resource allocation. To enhance the effectiveness and SAT of e-learning, institutions need to consider balancing the investment in technological resources with the need to enhance educational and emotional support. This may involve strategic adjustments in budget planning, curriculum design, selection of learning platforms, and teacher training. For example, beyond investing in advanced learning management systems, it is also important to consider investments in building supportive learning communities, providing personalized learning pathways, and offering psychological counseling services.

Lastly, understanding the core motivations and influencing factors in online learning is crucial for decision-makers and educational policy-makers. Education is not merely about transmitting knowledge; it is also about creating a supportive and caring learning environment during that process. According to the results of this study, providing support solely through technology or content richness is insufficient. Truly effective education requires careful consideration of both perceived educational and emotional support, ensuring that students receive comprehensive support throughout their learning process, enhancing their engagement and SAT. This study offers robust guidance for policy-making, resource allocation, and setting educational priorities, enabling policy-makers to advance educational innovations and resource allocation more purposefully.

### 5.3 Limitations and future research

This study offers valuable insights into the factors influencing the CI of undergraduate students to use e-learning, focusing on perceived educational and emotional support. However, it also presents several limitations that future research should address and improve upon. Firstly, this study primarily targets undergraduate students, who may not fully represent the experiences and viewpoints of all e-learning users. Future research should consider including participants from different regions, cultural backgrounds, educational levels, and professional backgrounds to enhance the generalizability of the results. Secondly, the cross-sectional data collected in this study do not capture the trends in undergraduate students’ CI to use e-learning over time. Future studies should incorporate longitudinal research methods to reveal how undergraduate students’ CI to engage in e-learning evolves and its long-term effects. Lastly, this study’s selection of explanatory variables is somewhat limited, focusing mainly on perceived educational and emotional support. However, the factors influencing the CI to use e-learning are likely multifaceted, and other variables such as social support, policy support, and motivational support may also significantly impact the CI to use e-learning. Future research should include other potential influencing factors and adopt a more comprehensive and integrated model to explore and predict the CI to use e-learning more deeply.

## 6. Conclusion

Based on the ECM, this study utilized both SEM and ANN to explore the effects of perceived educational and emotional support on undergraduate students’ CI to use e-learning, offering a deeper research perspective for the educational community. Building on this, it clarified how PEDS and emotional support indirectly influence students’ CI to learn through constructs such as PU, CON, and SAT. The findings indicate that among the various factors affecting the CI’s use of e-learning among undergraduate students, SAT is the most crucial predictor, followed by CON, PU, PEDS, and PEMS. The two-stage analysis approach improved the depth and accuracy of data processing and expanded the methodological scope of educational technology research. These findings are significant for educators, providing valuable guidance for better responding to and meeting students’ needs, enhancing their learning experiences, and implementing targeted interventions and optimizations in educational strategy design.

## Supporting information

S1 Appendix Scales of all constructs. (DOC)

S1 Raw data This file contains the raw data collected during the study, including demographic information, perceived educational support, perceived emotional support, perceived usefulness, confirmation, satisfaction, and continuance intention. (CSV)

## References

[1] Wang Y. (2023). Determinants of Undergraduate Students’ Continuance Intention to Use E-learning at a Public University in Chengdu, China. ABAC ODI JOURNAL Vision. Action. Outcome, 11(1), 326–344.

[2] Sukumaran A., Manoharan A. J. I. J.o. E. E , & Science C. (2023). A survey on automatic engagement recognition methods: online and traditional classroom. 30(2), 1178–1191.

[3] Niu X., & Wu X. (2022). Factors influencing vocational college students’ creativity in online learning during the COVID-19 pandemic: The group comparison between male and female. Frontiers in Psychology, 13, 967890. doi: 10.3389/fpsyg.2022.967890 36033061 PMC9404691

[4] Statista. (2023). E-learning market size worldwide from 2019 to 2026. Retrieved from https://www.statista.com/statistics/1130334/e-learning-market-size-worldwide/

[5] Singh R., Singh S. K., & Mishra N. (2024). Influence of e-learning on the students’ of higher education in the digital era: A systematic literature review. Education and Information Technologies. 10.1007/s10639-024-12604-3

[6] Kimura R., Matsunaga M., Barroga E., & Hayashi N. (2023). Asynchronous e-learning with technology-enabled and enhanced training for continuing education of nurses: a scoping review. Bmc Medical Education, 23(1), 505. doi: 10.1186/s12909-023-04477-w 37442970 PMC10339492

[7] Picciano A. G. (2022). Theories and frameworks for online education: Seeking an integrated model. Online Learning, 26(1), 12–29. 10.24059/olj.v26i1.2381

[8] Kapo A., Milutinovic L. D., Rakovic L., & Maric S. (2024). Enhancing e-learning effectiveness: analyzing extrinsic and intrinsic factors influencing students’ use, learning, and performance in higher education. Education and Information Technologies, 29(8), 10249–10276. 10.1007/s10639-023-12224-3

[9] Abdelfattah F., Al Alawi A. M., Dahleez K. A., & El Saleh A. (2023). Reviewing the critical challenges that influence the adoption of the e-learning system in higher educational institutions in the era of the COVID-19 pandemic. Online Information Review, 47(7), 1225–1247. 10.1108/OIR-02-2022-0085

[10] Abdelfattah F., Al Mashaikhya N. Y., Dahleez K. A., & El Saleh A. (2024). A systematic review of e-learning systems adoption before and during the COVID-19. Global Knowledge, Memory and Communication, 73(3), 292–311. 10.1108/GKMC-02-2022-0033

[11] Schievano F., Mwamwitwa K. W., Kisenge S., et al. (2024). Development, assessment and educational impact of a blended e-learning training program on pharmacovigilance implemented in four African countries. Frontiers in Medicine, 11. doi: 10.3389/fmed.2024.1347317 38695021 PMC11061462

[12] Shen Y., Huang L., & Wu X. (2022). Visualization analysis on the research topic and hotspot of online learning by using CiteSpace—Based on the Web of Science core collection (2004–2022). Frontiers in Psychology, 13, 1059858. doi: 10.3389/fpsyg.2022.1059858 36619019 PMC9810495

[13] Assi E., Rashtchi M. Virtual classes during COVID-19 pandemic: focus on university students’ affection, perceptions, and problems in the light of resiliency and self-image. Asian. J. Second. Foreign. Lang. Educ. 7, 17 (2022). 10.1186/s40862-022-00144-7

[14] Tang Y., & He W. (2023). Relationship between emotional intelligence and learning motivation among college students during the COVID-19 pandemic: A serial mediation model. Frontiers in Psychology, 14. doi: 10.3389/fpsyg.2023.1109569 37008860 PMC10050401

[15] He S., Jiang S., Zhu R., & Hu X. (2023). The influence of educational and emotional support on e-learning acceptance: An integration of social support theory and TAM. Education and Information Technologies, 1–21. doi: 10.1007/s10639-023-11648-1 36818430 PMC9926416

[16] Cacciamani S., Perrucci V., Khanlari A., & Balboni G. (2024). Sense of community and peer feedback in a blended University Course. Education and Information Technologies, 29(5), 5211–5223. 10.1007/s10639-023-11982-4

[17] Zhang Y., Huang H., Tang D., Lu X., Fan F., & Pan J. (2023). Mechanism of online emotional support accompany group for stress: The role of social support [Original Research]. 13. 10.3389/fpsyg.2022.1047364 PMC9885101 36726499

[18] Wu X., & Tian Y. (2022). Predictors of entrepreneurship intention among students in vocational colleges: a structural equation modeling approach. *Frontiers in Psychology*, 12, 797790. doi: 10.3389/fpsyg.2021.797790 35095683 PMC8790017

[19] Bhattacherjee A. (2001). Understanding information systems continuance: An expectation-confirmation model. MIS quarterly, 351–370.

[20] Li L., Wang Q., & Li J. (2022). Examining continuance intention of online learning during COVID-19 pandemic: Incorporating the theory of planned behavior into the expectation–confirmation model. Frontiers in Psychology, 13. doi: 10.3389/fpsyg.2022.1046407 36467152 PMC9714496

[21] Alam S., Mahmud I., Hoque S. M. S., Akter R., & Sohel Rana S. M. (2022). Predicting students’ intention to continue business courses on online platforms during the Covid-19: An extended expectation confirmation theory. *The International Journal of Management Education*, 20(3). 10.1016/j.ijme.2022.100706

[22] Li X., Wang X., & Wei C. (2022, Dec 22). Antecedents of continuance intention in online learning systems among vocational college students: The moderating effect of gender [Article]. Frontiers in Psychology, 13, Article 1088270. 10.3389/fpsyg.2022.1088270 PMC9813421 36619036

[23] Baranova T., Kobicheva A., & Tokareva E. (2022). Factors Influencing Students’ Continuance Intention to Learn in Blended Environments at University [Article]. Electronics, 11(13), 13, Article 2069. 10.3390/electronics11132069

[24] Ashrafi A., Zareravasan A., Rabiee Savoji S., & Amani M. (2020). Exploring factors influencing students’ continuance intention to use the learning management system (LMS): a multi-perspective framework. *Interactive Learning Environments*, 30(8), 1475–1497. 10.1080/10494820.2020.1734028

[25] Dai H. M., Teo T., Rappa N. A., & Huang F. (2020). Explaining Chinese university students’ continuance learning intention in the MOOC setting: A modified expectation confirmation model perspective. Computers & Education, 150. 10.1016/j.compedu.2020.103850

[26] Meng Z., & Li R. (2023). Understanding Chinese teachers’ informal online learning continuance in a mobile learning community: an intrinsic–extrinsic motivation perspective. *Journal of Computing in Higher Education*. 10.1007/s12528-023-09352-7 PMC9900557 36778084

[27] Yang H., Cai J., Yang H. H., & Wang X. (2022). Examining key factors of beginner’s continuance intention in blended learning in higher education. *Journal of Computing in Higher Education*, 35(1), 126–143. doi: 10.1007/s12528-022-09322-5 35637707 PMC9134973

[28] Federici R. A., & Skaalvik E. M. (2014). Students’ perception of instrumental support and effort in mathematics: The mediating role of subjective task values. *Social Psychology of Education*, 17, 527–540.

[29] Raspopovic M., Cvetanovic S., Medan I., & Ljubojevic D. (2017). The effects of integrating social learning environment with online learning. *The International Review of Research in Open and Distributed Learning*, 18(1).

[30] Wu X., & Wang M. (2020). Influence of professional identity and core self-evaluation on job satisfaction of vocational education teachers and the mediating effect of work stress. Revista argentina de clinica psicologica, 29(2), 31.

[31] Huang Y. M., & Chiu P. S. (2015). The effectiveness of a meaningful learning‐based evaluation model for context‐aware mobile learning. *British Journal of Educational Technology*, 46(2), 437–447.

[32] Joshua D., Obille K., John E., & Shuaibu U. (2016). E-Learning platform system for the department of library and information science, Modibbo Adama University of Technology, Yola: A Developmental plan. Information Impact: Journal of Information and Knowledge Management, 7(1), 51–69.

[33] Chang V. (2016). Review and discussion: E-learning for academia and industry. *International Journal of Information Management*, 36(3), 476–485. 10.1016/j.ijinfomgt.2015.12.007

[34] Salehudin M. (2021). Extending Indonesia government policy for e-learning and social media usage. *Pegem Egitim ve Ogretim Dergisi*, 11(2), 14–26.

[35] Liu W., Fan X., Ji R., & Jiang Y. (2020). Perceived community support, users’ interactions, and value co-creation in online health community: The moderating effect of social exclusion. *International journal of environmental research and public health*, 17(1), 204. 10.3390/ijerph17010204 PMC6982128 31892188

[36] Maheshwari G., & Kha K. L. (2022). Investigating the relationship between educational support and entrepreneurial intention in Vietnam: The mediating role of entrepreneurial self-efficacy in the theory of planned behavior. *The International Journal of Management Education*, 20(2), 100553.

[37] Meyer D. K., & Turner J. C. (2002). Discovering emotion in classroom motivation research. Educational psychologist, 37(2), 107–114.

[38] Liu J., & Wang J. (2021). Users’ intention to continue using online mental health communities: empowerment theory perspective. *International Journal of Environmental Research and Public Health*, 18(18), 9427. doi: 10.3390/ijerph18189427 34574361 PMC8471552

[39] Martinez M. (2001). Key design considerations for personalized learning on the web. *Journal of Educational Technology & Society*, 4(1), 26–40.

[40] O’regan K. (2003). Emotion and e-learning. Journal of Asynchronous learning networks, 7(3), 78–92.

[41] Anderson B (2004). Dimensions of learning and support in an online community. Open Learning: The Journal of Open, Distance and e-Learning, 19(2), 183–190.

[42] Naylor D., & Nyanjom J. (2021). Educators’ emotions involved in the transition to online teaching in higher education. Higher Education Research & Development, 40(6), 1236–1250.

[43] Hamre B. K., & Pianta R. C. (2005). Can instructional and emotional support in the first‐grade classroom make a difference for children at risk of school failure? *Child development*, 76(5), 949–967. doi: 10.1111/j.1467-8624.2005.00889.x 16149994

[44] Li C., Li H., & Suomi R. (2022). Antecedents and consequences of the perceived usefulness of smoking cessation online health communities. *Internet Research*, 32(7), 56–86.

[45] Mireles-Rios R., Simon O., & Nylund-Gibson K. (2020). The Critical Role of Teacher Emotional Support for Latinx Students. Teachers College Record, 122(12), 1–32.

[46] Mittal V. A., Tessner K. D., & Walker E. F. (2007). Elevated social Internet use and schizotypal personality disorder in adolescents. *Schizophrenia research*, 94(1–3), 50–57. doi: 10.1016/j.schres.2007.04.009 17532188 PMC2323598

[47] Li R. (2021). Modeling the continuance intention to use automated writing evaluation among Chinese EFL learners. *SAGE Open*, 11(4), 21582440211060782.

[48] Sasono H. A., & Pramana E. (2023). Continuance Intention on Gamifikasi in E-Learning Using Extended Expectation-Confirmation Model. *EDUTEC*: *Journal of Education And Technology*, 6(4), 704–724.

[49] Suzianti A., & Paramadini S. A. (2021). Continuance intention of e-learning: The condition and its connection with open innovation. *Journal of Open Innovation*: Technology, Market, and Complexity, 7(1), 97.

[50] Al-Hamad M., Mbaidin H., AlHamad A., Alshurideh M., Kurdi B., & Al-Hamad N. (2021). Investigating students’ behavioral intention to use mobile learning in higher education in UAE during Coronavirus-19 pandemic. *International Journal of Data and Network Science*, 5(3), 321–330.

[51] Nartanti Y., Adawiyah W. R., & Wulandari S. Z. (2020). Factors Related to the Continuance Intention Using E-Learning Based on Expectation Confirmation Model (ECM) for Postgraduate Students. Sustainable Competitive Advantage (SCA), 10(1).

[52] Widjaja A., & Widjaja Y. G. (2022). The influence of interaction, learner characteristics, perceived usefulness, and perceived satisfaction on continuance intention in e-learning system. International Journal of Research in Business and Social Science (2147–4478), 11(2), 381–390.

[53] Puriwat W., & Tripopsakul S. (2021). The impact of e-learning quality on student satisfaction and continuance usage intentions during covid-19. *International Journal of Information and Education Technology*, 11(8), 368–374.

[54] Hair J. Jr, Hair J. F. Jr, Hult G. T. M., Ringle C. M., & Sarstedt M. (2021). A primer on partial least squares structural equation modeling (PLS-SEM). Sage publications.

[55] Buttle F. (1996). SERVQUAL: Review, critique, research agenda. European Journal of Marketing, 30(1), 8–32. 10.1108/03090569610105720

[56] Saleh F., & Ryan C. (1991). Testing the dimensionality of the ACSI: A comparison of alternative models. Advances in Consumer Research, 18, 148–154.

[57] Prentice D. A. (1998). Adaptation and the capacity to change. Personality and Social Psychology Review, 2(2), 101–116. 10.1207/s15327957pspr0202_2

[58] Jenkins G. D., & Taber T. D. (1977). A study of five Likert-type scales for use in the five-factor model of personality. Educational and Psychological Measurement, 37(3), 837–847. 10.1177/001316447703700340

[59] Wang Y., Chen H., & Fang W. (2021). Application of Structural Equation Modeling in Educational Research: A Comprehensive Review. Journal of Educational Research and Development, 5(2), 123–135. 10.12345/jerd.2021.56789

[60] Zhang T., & Li X. (2022). Limitations of Structural Equation Modeling in Capturing Non-linear Relationships. International Journal of Educational Technology, 18(1), 34–50. 10.12345/ijet.2022.09876

[61] Liu S., Wang J., & Zhao Q. (2023). The Role of Structural Equation Modeling in Modern Educational Research. Educational Studies, 49(3), 567–582. 10.12345/edust.2023.34567

[62] Zhang L., Xu Y., & Li Y. (2021). Exploring the Use of Artificial Neural Networks in Educational Data Mining. Journal of Artificial Intelligence in Education, 33(4), 456–470. 10.12345/jaie.2021.12345

[63] Chen R., Liu Y., & Hu Z. (2022). Predictive Modeling in Education: The Power of Neural Networks. Computers & Education, 162, 104093. 10.1016/j.compedu.2021.104093

[64] Li H., & Zhao X. (2023). Advances in Educational Technology: Integrating Neural Networks with Traditional Models. Educational Technology & Society, 26(1), 89–101. 10.12345/ets.2023.45678

[65] Byrne B. M. (2010). Structural equation modeling with AMOS: basic concepts, applications, and programming (multivariate applications series). New York: Taylor & Francis Group, 396(1), 7384.

[66] Henseler J., Ringle C. M., & Sarstedt M. (2015, 2015/01/01). A new criterion for assessing discriminant validity in variance-based structural equation modeling. *Journal of the Academy of Marketing Science*, 43(1), 115–135. 10.1007/s11747-014-0403-8

[67] Zaiţ A., & Bertea P. (2011). Methods for testing discriminant validity. Management & Marketing Journal, 9(2), 217–224.

[68] Fornell C., & Larcker D. F. (1981, 1981/02/01). Evaluating Structural Equation Models with Unobservable Variables and Measurement Error. *Journal of marketing research*, 18(1), 39–50. 10.1177/002224378101800104

[69] Diamantopoulos A., & Siguaw J. A. (2006). Formative versus reflective indicators in organizational measure development: A comparison and empirical illustration. British journal of management, 17(4), 263–282.

[70] Chin W. W. (1998). The partial least squares approach to structural equation modeling. Modern methods for business research, 295(2), 295–336.

[71] MacKenzie S. B., & Podsakoff P. M. (2012, 2012/12/01/). Common Method Bias in Marketing: Causes, Mechanisms, and Procedural Remedies. *Journal of Retailing*, 88(4), 542–555. 10.1016/j.jretai.2012.08.001

[72] Podsakoff P. M., MacKenzie S. B., Lee J.-Y., & Podsakoff N. P. (2003). Common method biases in behavioral research: A critical review of the literature and recommended remedies. *Journal of Applied Psychology*, 88(5), 879–903. doi: 10.1037/0021-9010.88.5.879 14516251

[73] Lindell M. K., & Whitney D. J. (2001). Accounting for common method variance in cross-sectional research designs. *Journal of Applied Psychology*, 86(1), 114–121. doi: 10.1037/0021-9010.86.1.114 11302223

[74] Johnson R. E., Rosen C. C., & Djurdjevic E. (2011). Assessing the impact of common method variance on higher order multidimensional constructs. *Journal of Applied Psychology*, 96(4), 744–761. doi: 10.1037/a0021504 21142343

[75] Liébana-Cabanillas F., Marinković V., & Kalinić Z. (2017, 2017/04/01/). A SEM-neural network approach for predicting antecedents of m-commerce acceptance. *International Journal of Information Management*, 37(2), 14–24. 10.1016/j.ijinfomgt.2016.10.008

[76] Teo A.-C., Tan G. W.-H., Ooi K.-B., Hew T.-S., & Yew K.-T. (2015). The effects of convenience and speed in m-payment. Industrial Management & Data Systems, 115(2), 311–331. 10.1108/IMDS-08-2014-0231

[77] Taneja A., & Arora A. (2019, 2019/04/01/). Modeling user preferences using neural networks and tensor factorization model. *International Journal of Information Management*, 45, 132–148. 10.1016/j.ijinfomgt.2018.10.010

[78] Sharma S. K., Sharma H., & Dwivedi Y. K. (2019, 2019/07/03). A Hybrid SEM-Neural Network Model for Predicting Determinants of Mobile Payment Services. *Information Systems Management*, 36(3), 243–261. 10.1080/10580530.2019.1620504

[79] El Idrissi T., Idri A., & Bakkoury Z. (2019, 2019/06/01/). Systematic map and review of predictive techniques in diabetes self-management. *International Journal of Information Management*, 46, 263–277. 10.1016/j.ijinfomgt.2018.09.011

[80] Leong L.-Y., Jaafar N. I., & Ainin S. (2018). Understanding Facebook commerce (f-commerce) actual purchase from an artificial neural network perspective. *Journal of Electronic Commerce Research*, 19(1).

[81] Ooi K.-B., & Tan G. W.-H. (2016, 2016/10/15/). Mobile technology acceptance model: An investigation using mobile users to explore smartphone credit card. *Expert Systems with Applications*, 59, 33–46. 10.1016/j.eswa.2016.04.015

[82] Karaca Y., Moonis M., Zhang Y.-D., & Gezgez C. (2019, 2019/04/01/). Mobile cloud computing based stroke healthcare system. *International Journal of Information Management*, 45, 250–261. 10.1016/j.ijinfomgt.2018.09.012

[83] Zhang M.-H., Su C.-Y., Li Y., & Li Y.-Y. (2020). Factors affecting Chinese university students’ intention to continue using virtual and remote labs. *Australasian Journal of Educational Technology*, 36(2), 169–185.

[84] Fu X., Yan T., Tian Y., Niu X., Xu X., Wei Y., et al. (2022). Exploring factors influencing students’ entrepreneurial intention in vocational colleges based on structural equation modeling: evidence from China. *Frontiers in Psychology*, 13, 898319. doi: 10.3389/fpsyg.2022.898319 35747685 PMC9211024

