# Supplementary material for: Hybrid SEM-ANN model for predicting undergraduates’ e-learning continuance intention based on perceived educational and emotional support
Source: PLoS One. 2024 Dec 13;19(12):e0308630. doi: 10.1371/journal.pone.0308630 (PMC11643250; doi:10.1371/journal.pone.0308630)
Supplement: S1 Appendix — (DOC) [file pone.0308630.s001.doc]

S1 Appendix. Scales of all constructs

| Construct | items | Source |
| --- | --- | --- |
| PEdS | When I use e-learning, my peers provide information, suggestions, and guidance. | Federici and Skaalvik (2014),Weng et al. (2015) |
|  | When I use e-learning, my teacher provides information and helps me improve efficiency. |  |
|  | When I have questions or doubts about e-learning, my teacher assists me. |  |
|  | When I encounter difficulties in e-learning, I can always seek help from my peers. |  |
| PEmS | When I use e-learning, my peers encourage and praise me. | Malecki and Demaray (2003),Tan et al. (2019),Weng et al. (2015) |
|  | When I face challenges in e-learning, my teacher is willing to listen and provide the emotional support I need. |  |
|  | My good friend kindly tells me the truth about how I perform. |  |
|  | My teacher kindly informed me about my genuine performance. |  |
| PU | I believe that using e-learning can improve my academic performance. | Kim et al. (2010),Wu and Chen (2017),Wu and Zhang (2014) |
|  | I think that using e-learning can enhance my study efficiency. |  |
|  | I feel that using e-learning makes it easy to transform learning materials into concrete knowledge. |  |
| CON | My experience with e-learning exceeded my expectations. | Rajeh et al. (2021),Rohan et al. (2021) |
|  | The services provided by e-learning exceeded my expectations. |  |
|  | Most of my expectations for e-learning have been confirmed. |  |
| SAT | I would recommend e-learning to others. | Jung and Shin (2021),Sumi and Kabir (2021) |
|  | My decision to choose e-learning was right. |  |
|  | I am very satisfied with the use of the electronic learning system. |  |
| CI | I will use e-learning regularly in the future. | Wu and Chen (2017),Wu and Zhang (2014) |
|  | I will use e-learning frequently in the future. |  |
|  | I will use e-learning more and more in the future. |  |
